# The impact of COVID-19 on students’ anxiety and its clarification: a systematic review

**DOI:** 10.3389/fpsyg.2023.1134703

**Published:** 2023-08-24

**Authors:** Jiarun Wu, Garry Kuan, Hu Lou, Xiaoyu Hu, Mohamad Najmi Masri, Abdulwali Sabo, Yee Cheng Kueh

**Affiliations:** ^1^Exercise and Sports Science Programme, School of Health Sciences, Universiti Sains Malaysia, Kota Bharu, Kelantan, Malaysia; ^2^School of Physical Health, Guizhou University of Traditional Chinese Medicine, Guiyang, Guizhou, China; ^3^School of Sports Science, Nantong University, Nantong, Jiangsu, China; ^4^Dafang County No. 7 Middle School, Bijie, Guizhou, China; ^5^Faculty of Bioengineering and Technology, Universiti Malaysia Kelantan, Kota Bharu, Kelantan, Malaysia; ^6^Biostatistics and Research Methodology Unit, School of Medical Sciences, Universiti Sains Malaysia, Kota Bharu, Kelantan, Malaysia

**Keywords:** COVID-19, stress, anxiety, depression, coping

## Abstract

**Introduction:**

Since the emergence of COVID-19 in 2019, every country in the world has been affected to varying degrees. Long-term psychological pressure and anxiety will inevitably damage the physical and mental health of students. This study aimed to examine the effects of the COVID-19 pandemic on students who experienced stress and anxiety and to clarify which intervention was more effective.

**Methods:**

A comprehensive literature search was conducted between January 2020 and December 2022 using online databases such as PubMed, Web of Science, Scopus, and Google Scholar by using the following keywords in combination: “COVID-19,” “stress,” “anxiety,” “depression,” and “intervention.” The retrieved literature was screened and reviewed.

**Results:**

A total of 2,924 articles were retrieved using subject and keyword searches. After screening through the titles and abstracts, 18 related studies were retained. Their review revealed that: (1) most studies did not use medication to control stress and anxiety; (2) the standard methods used to reduce stress and anxiety were religion, psychological counseling, learning more about COVID-19 through the media, online mindfulness courses, improving sleep quality, and physical exercise; (3) the most effective interventions were physical activity and raising awareness about COVID-19 through the media and online mindfulness programs. However, some studies show that physical activity cannot directly relieve psychological stress and anxiety.

**Conclusion:**

Limited interventions are effective, but learning more about COVID-19 and using active coping strategies may help reduce stress and anxiety. The implications of COVID-19 are also discussed.

## Introduction

Since the COVID-19 outbreak in December 2019, the virus has maintained exceptionally high transmission rates, and countries around the world have been greatly affected. According to the World Health Organization’s (WHO) COVID-19 Dashboard (2022), the cumulative number of confirmed cases worldwide exceeded 600 million as of September of that same year. Due to the continuous mutation of COVID-19, the number of confirmed and suspected cases remains high, and people are forced to live with the virus for a long time, which creates a lot of psychological anxiety and stress and seriously affects the normal lives of people around the world (Peteet, 2020).

Although the severity of COVID-19 has decreased over time and the current level of prevention and control of the epidemic in the world has also decreased, COVID-19 has not been eliminated and still poses a threat to humanity (World Health Organization, 2023a,b). As noted in the WHO’s most recent official COVID-19 policy brief, the pandemic appears to be in transition after more than 3 years. However, the COVID-19 pandemic remains an acute global emergency, with the risk of the emergence of new variants and future surges remaining real (World Health Organization, 2023a,b).

According to data released by the WHO COVID-19 Dashboard of 2023, as of 7 June, there had been 6,941,095 deaths and 767,750,853 confirmed cases of COVID-19 worldwide. Furthermore, according to the WHO Coronavirus (COVID-19) Dashboard, the COVID-19 outbreak has shown unpredictable volatility in terms of weekly changes in the number of confirmed cases worldwide every week since the outbreak began, with the most recent large-scale outbreak appearing in December 2022. In just 4 weeks, the outbreak increased the number of cases worldwide by 97,976,070 (World Health Organization, 2022a,b).

It is clear from these findings that COVID-19 remains a threat to humans. To deal with the possibility of a recurrence of the virus pandemic in the future, we need to understand the various effects of COVID-19 on people’s psychological states and develop effective responses.

Many psychological effects have been examined during the virus outbreak at the personal, national, and even international levels (Fawaz et al., 2023). On a personal level, people are more likely to experience fear of getting sick or dying, feeling helpless, and being stereotyped by others (Salari et al., 2020). Mental health can contribute to serious psychological crises. In fact, COVID-19 affected the mental health of medical workers and also the general population (Chakraborty and Chatterjee, 2020; Khatatbeh et al., 2021; Chen et al., 2022). For healthcare workers, the sudden increase in workload has led to a surge in work stress (Tabur et al., 2022), and the risk of infection at any time has had a negative impact on their psychology (Wu et al., 2020). Witnessing the suffering and death of patients can also have a profound impact on their mental health (Mosheva et al., 2021). In addition, the uncertainty and rapid change of the COVID-19 pandemic mean that they must constantly adapt to new job requirements, changing work processes, and protective measures, and the stress caused by this uncertainty may also increase their anxiety levels (Deliktas Demirci et al., 2021). For the general population, COVID-19 may increase health anxiety and panic, and concern over the risk of infection may cause people to over-interpret common physiological responses (Marra et al., 2020). Reduced human interaction due to fear of infection can also lead to depression and anxiety (Haider et al., 2020). In addition, financial stress and work stress also contribute to a decline in mental health (Halliburton et al., 2021). The WHO has also reported that the COVID-19 pandemic triggered a 25% increase in the prevalence of anxiety and depression worldwide (World Health Organization, 2022a,b).

University students face certain peculiarities compared to working adults. They will show emotional instability under pressure. Their psychological characteristics differ from those of the general public or even ordinary young people, and they are high-risk groups for psychological problems (Kontoangelos et al., 2020; Fusar-Poli et al., 2021). Under the influence of an external environment, psychological problems such as anxiety, depression, and post-traumatic stress disorder (PTSD) are more likely to occur (Bruffaerts et al., 2018). They face greater academic pressures and challenges, and their worries about academic performance and work prospects are more intense (Son et al., 2020). Compared with adults, they are more susceptible to the adverse effects of distance learning due to their lack of experience in time management (Singh et al., 2020). Most of the students social and interpersonal relationships exist on campus, and COVID-19 isolation has cut off most of their social ties, increased their loneliness, and led to a decline in mental health. This will last for a period of time and will not disappear quickly with the end of the epidemic (Garris and Fleck, 2022). In addition, in terms of independence and social identity, COVID-19 can also have a negative impact on independence and social identity and last for a long time (Sharaievska et al., 2022).

How to deal with the psychological problems faced by university students due to the COVID-19 epidemic has become a crucial research topic at this stage (González-Sanguino et al., 2020). The purpose of this article is to review articles on the different interventions applied to reduce the psychological impact of COVID-19 and to discuss solutions among students. First of all, such research has a long-term and global impact. A large number of studies have confirmed that COVID-19 has broad psychological effects on university students worldwide. Understanding the long-term impact of COVID-19 on the mental health of university students and adaptation strategies can provide experience and lessons for similar emergencies in the future (De Girolamo et al., 2020). Second, by evaluating the effectiveness of different mental health intervention strategies, we can determine which interventions have a positive impact on alleviating mental distress and improving the mental health of university students, which can help guide mental health professionals and policymakers in choosing appropriate intervention strategies to better support their mental health. In addition, by focusing on the mental health of college students, it is helpful to increase public awareness and attention to the mental health of college students and promote overall societal attention to mental health (Hunt and Eisenberg, 2010). There are three problems to be solved in this study: (a) To summarize the effects of COVID-19 on the psychological status of students; (b) To compare the impact of different interventions; (c) To discuss the findings and recommendations for future research in this area.

## Methodology

### Search strategy

A literature search was conducted using major computerized databases (e.g., PubMed, Web of Science, Scopus, and Google Scholar) and library holdings for English-language peer-reviewed articles and was reviewed by two additional co-authors. When searching the database, the selected studies were those published from the beginning of January 2020 to December 2022 (because the outbreak of COVID-19 began in December 2019, this study only looked at studies over a three-year period, from January 2020 to December 2022, when searching the databases). The keywords used in this review were “COVID-19,” “anxiety,” “stress,” “student,” and “intervention.” A manual search of the reference lists of relevant studies found in the computerized search was also performed.

### Inclusion and exclusion criteria

**Figure 1:**
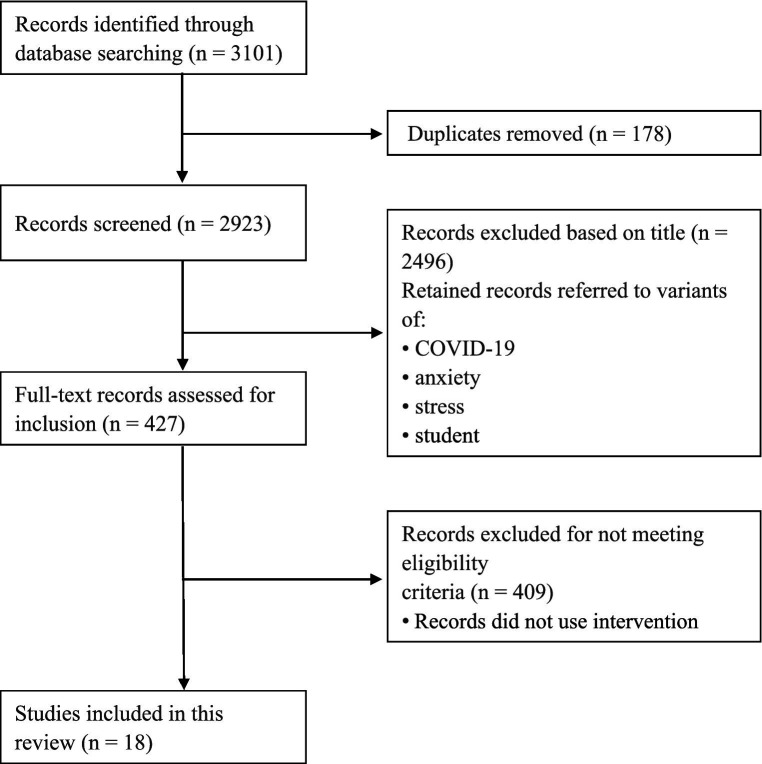
Flow diagram from identification to inclusion of studies.

The inclusion of articles followed a three-phase approach (Figure 1) using the Preferred Reporting Items for Systematic Reviews and Meta-Analyses (PRISMA) guidelines (Moher et al., 2015). In the first phase, a total of 3,101 records were initially obtained through an extensive database search. A total of 178 duplicates were identified and removed in this phase. In the second phase, the titles of 2,923 records were screened, and the following records were removed: (a) those that did not refer to the terms “COVID-19” or “anxiety” or “stress” or “student”; (b) all types of literature reviews and guidelines; and (c) those that were not written in English. This process resulted in the removal of 2,496 records. In the third phase, the abstracts or full texts of the remaining 427 records were examined. Records that met the inclusion criteria were studies with an interventional design. Only 18 studies met the inclusion criteria, and the authors and co-authors assessed the quality of these 18 studies and found them to be of good quality for a systematic review and were therefore included in this review.

### Categorization of studies

## Results

### The impact of COVID-19 on the psychological status of students

#### Research on the psychological status of medical college students

Early in the COVID-19 outbreak, the Association of American Medical Colleges recommended pausing all student clinical rotations while the in-person curriculum moved to virtual modalities. Medical students also reported higher levels of anxiety, stress, and exhaustion, with female students experiencing this more than male students (Mittal et al., 2021). At the College of Medicine of King Saud University, Riyad, Saudi Arabia, a total of 234 medical students found that quarantine caused them to feel emotionally detached from family, colleagues, and friends and reduced their overall work performance and study time. The findings also showed that a quarter of the medical students who participated in the study felt disheartened during the quarantine period (Meo et al., 2020). In a study of 217 undergraduate medical students at a medical college in Chennai, India, there was a significant increase in both the prevalence and level of anxiety and stress (Saraswathi et al., 2020). In an Indonesian study of 1,027 medical students, 44.6% were found to be stressed, 47.8% had anxiety, and 18.6% had depression (Natalia and Syakurah, 2021).

#### Research on the mental state of the general student population

The psychological state of the general student population has also been a focus of researchers during the COVID-19 pandemic. A study of Bangladesh university students aged 18 to 29 years (59.5% men; mean age 21.4 ± 2 years) revealed that the estimated prevalence of depression, anxiety, and stress was 76.1, 71.5, and 70.1% for at least mild symptoms, 62.9, 63.6, and 58.6% for at least moderate symptoms, 35.2, 40.3, and 37.7% for at least severe symptoms, and 19.7, 27.5, and 16.5% for at least very severe symptoms, respectively. These data are significantly higher than before the COVID-19 pandemic (Islam et al., 2020). In a cross-sectional survey of different populations in Jordan, anxiety was highest among college students during the COVID-19 pandemic, at 21.5%, and college students were found to be at higher risk for depression (Naser et al., 2020). In another study of 69,054 high school students in France, the prevalence of suicidal thoughts, severe distress, high levels of perceived stress, severe depression, and high levels of anxiety were 11.4% (7,891 students), 22.4% (15,463 students), 24.7% (17,093 students), 16.1% (11,133 students), and 27.5% (18,970 students), respectively (Wathelet et al., 2020). Similarly, a study of 1,224 high school students in Brazil reported that the majority of undergraduates presented with symptoms of depression (60.5%), anxiety (52.5%), and stress (57.5%). These data indicated a high prevalence of symptoms of depression, anxiety, and stress in students (Lopes and Nihei, 2021). In summary, numerous studies have reported on the mental health of students during the COVID-19 pandemic, and the results show that the pandemic has caused varying degrees of mental health problems among students, indicating that the presence of COVID-19 has indeed increased psychological stress and anxiety among the student population around the world.

#### The effects of the different interventions

Since the outbreak of COVID-19, some research studies have tried various ways to reduce people’s stress and anxiety. By collating the existing literature, the primary approaches for reducing stress and anxiety include religious practice, psychological counseling, learning more about COVID-19 through the media, mindfulness online programs, increasing the quality of sleep, and physical activity. Some of the most effective interventions include watching brief Dialectical Behavior Therapy (DBT) skill videos, regular web-based physical education to increase students’ understanding of COVID-19 through social media, and about 2,500 METs of physical activity per week (Deng et al., 2020; Zhang et al., 2020; Kheirallah et al., 2021; Rizvi et al., 2022). Although the effect of some intervention methods is not significant, they play a role as effective mediating factors. These methods include: middle- and long-distance running, boosting satisfaction with distance learning, strengthening psychological resilience, enhancing social support, improving sleep quality, reducing incorrect smartphone use, and catalyzing academic satisfaction (Franzen et al., 2021; Li and Peng, 2021; Lin et al., 2021; Liu et al., 2021; Gabrovec et al., 2022; Song et al., 2022). The intervention had positive results, and effective alternatives to conventional psychotherapy included behavioral therapy and online mindfulness programs (Liang et al., 2021; Simonsson et al., 2021). Some studies have suggested that positive approach coping strategies for COVID-19 are better than negative avoidance coping strategies in improving stress and anxiety (Banstola et al., 2021; Chan et al., 2022). A review of the literature identified 18 articles related to COVID-19, anxiety, stress, and the effects of different interventions (all studies are summarized in Table 1).

**Table 1:** Literature on different interventions evaluated in this study.

Study	Participants	Instrumentation/procedure	Main outcomes
Chan et al. (2022)	A total of 202 university students in Hong Kong, 70.8% of whom were medical students	(1) Basic information about stress and psychological disorders, (2) The Ryff Scale (18 items), (3) Brief list of coping skills.	The Ryff score of the students who used proximity coping strategies was higher than that of the students who used avoidance coping strategies. Proximity coping strategies are more effective in managing stress.
Banstola et al. (2021)	A total of 144 students at the Manipal College of Medical Sciences in Pokhara, Nepal	Clinical and COVID-19 related questions; Beck Anxiety Inventory; Brief-COPE questionnaire to assess coping strategies.	The most common coping strategy was religion; adopting adaptive coping strategies helps reduce mental health problems.
Rizvi et al. (2022)	A total of 153 U.S. undergraduates. They were randomly divided into an intervention group (*n* = 99) and a control group (*n* = 54)	(1) Demographic survey, (2) Difficulties in Emotion Regulation Scale-18, (3) Ecological Momentary Assessment (EMA), (4) Intervention via brief Dialectical Behavior Therapy (DBT) skill videos.	Four assessments per day. After the intervention, negative emotions were significantly reduced, while positive emotions were significantly increased. Increased emotional tolerance. DBT technology videos can help college students avoid mental health deterioration.
Lin et al. (2021)	A total of 869 middle- and long-distance running students at a university in Guangzhou, China	(1) Civilian version of the PTSD questionnaire (PCL-C), (2) Self-rating Anxiety Scale (SAS), (3) Civilian version of the PTSD Questionnaire (PCL-C). (4) Self-rating Depression Scale (SDS).	Middle and long-distance running exercise has no direct effect on students’ PTSD, anxiety, and depression, but it does have an effect on students’ body scores. At the same time, body score has an effect on students’ PTSD, anxiety, and depression. Furthermore, it played an interconnected role in promoting the mental health of students during the epidemic.
Deng et al. (2020)	A total of 1,607 college students in Wuhan, China, including 1,041 men and 566 women	Mental health status was assessed using the Depression, Anxiety, and Stress Scale (DASS-21).	Participants’ mean scores on the DASS-21 subscale after the intervention were significantly lower than in the previous study. Lower DASS-21 scores were significantly associated with an active exercise-related lifestyle.
Gabrovec et al. (2022)	A total of 4,661 Slovenian post-secondary students, with 72.5% women, 26.7% men, and 0.8% identifying as another gender.	(1) Patient Health Questionnaire Generalized Anxiety Disorder questionnaire, (2) Perceived Stress Scale-4 10-item Connor–Davidson Resilience Scale, (3) Satisfaction with Online Study Scale (SAT-5)-5	Distance learning satisfaction and resilience can be used as protective factors to influence students’ mental health. Increasing distance learning satisfaction and enhancing psychological resilience can indirectly reduce stress, anxiety, and depression. Female students need greater mental resilience to combat negative mental states.
Jain et al. (2021)	A total of 699 Indian university students, with 239 women and 460 men.	Mental health status was determined using the Coronavirus Anxiety Screening (CAS), GHQ-12, GAD-7, and PHQ-9 scales.	The prevalence of self-medication was found to be very low as compared to other studies, and a growing tendency towards homemade remedies was noted. This inclination is expected to come out as endorsements and guidelines of homemade remedies going around these days.
Li and Peng (2021)	A total of 2,640 college students in China	(1) Sociodemographic characteristics questionnaire (SCQ), (2) Coping strategy questionnaire (CSQ), (3) Social support questionnaire (SSQ), (4) Self-rating anxiety scale (SAS).	Anxiety was negatively associated with coping and social support. Social support played as a mediator in the relationships between cognitive coping, behavioral coping, and anxiety, with family support and counselor support exerting a stronger negative influence against anxiety than subjective support.
Liang et al. (2021)	A total of 52 students from Anhui Medical University in China, divided into two groups of 26 respondents, with 10 men and 16 women.	(1) PHQ-9 scale, (2) GAD-7 scale, (3) Somatic Self-rating Scale (SSS), (4) Perceived Stress Scale (PSS-10).	Dialectical behavior therapy (DBT) was more effective than traditional psychological intervention. DBT could effectively alleviate the depression and anxiety of medical students during the normalization of epidemic prevention and control.
Liu et al. (2021)	A total of 29,663 medical students in China, with 10,185 men (34.3%) and 19,478 women (65.7%)	(1) Perceived Stress Scale 14 (PSS-14), (2) Insomnia Severity Index (ISI), (3) Patient Health Questionnaire 9 (PHQ-9).	Perceived stress was associated with depression, and insomnia played a mediating role when included in the association. Interventions or strategies that improve insomnia may help reduce the severity of depression both directly and indirectly in medical students.
Seffrin et al. (2022)	A total of 40 students at the Federal University of São Paulo, with 10 men and 30 women	(1) Patient Health Questionnaire 9 (PHQ-9), (2) General Anxiety Disorder-7 (GAD-7).	Returning to online classes may mitigate the high frequency of depression symptoms observed during the social distancing measures adopted during the outbreak of COVID-19.
Simegn et al. (2021)	A total of 423 Ethiopian university students, with 272 men and 151 women	Depression, Anxiety, and Stress Scale-21	Mental health could be improved by the provision of adequate and accurate information and by increasing the self-efficacy of students.
Simonsson et al. (2021)	A total of 177 students from the University of Oxford	Outcome Measurement Information System (PROMIS) anxiety and depression scales	Participants randomized to mindfulness programs showed a greater reduction in anxiety after 8 weeks of intervention.
Song et al. (2022)	A total of 666 medical college students in Shenyang, China, with 262 men and 404 women	(1) GAD-7, (2) Smartphone addiction scale–short version (SAS-SV), (3) PROMIS Sleep Disturbance scale (short form)	Smartphone addiction may increase the likelihood of experiencing sleep disturbances, which in turn may lead to elevated levels of anxiety.
Franzen et al. (2021)	A total of 433 students from the University of Geneva with 76 men and 357 women.	(1) Hospital Anxiety and Depression Scale (HADS), (2) 14-item Cohen Perceived Stress Scale (PSS), (3) Psychological Well-Being Scale (BEP), (4) Scale of Satisfaction with Studies (SSS).	Compared to COVID-19-related stress, academic satisfaction was a stronger predictor of depression, anxiety, stress, and psychological well-being among students at the end of the academic year.
Yildirim et al. (2021)	A total of 115 Turkish university students, with 23 men and 92 women	Death Anxiety Scale (DAS)	This would be an effective method to provide training to nursing students, in order to change their negative attitudes and increase their awareness of COVID-19-related death anxiety. This training would also improve their coping skills for dealing with death anxiety and reduce the burden of anxiety.
Zhang et al. (2020)	A total of 66 college students in China, with 25 men and 41 women	(1) Short version of the International Physical Activity Questionnaire (IPAQ-S), (2) Pittsburgh Sleep Quality Index (PSQI), (3) DASS-21, (4) Buss-Perry Aggressive Questionnaire (BPAQ).	The COVID-19 death toll has had an indirect impact on general negative emotions, stress, and anxiety, with sleep quality acting as a mediator. Moreover, physical activity directly alleviated general negative emotions, and the maximum mitigation effect occurred when weekly physical activity reached approximately 2,500 METs.

Of the 18 articles included, four showed significant effects on students’ stress and anxiety after the intervention (Deng et al., 2020; Zhang et al., 2020; Kheirallah et al., 2021; Rizvi et al., 2022). In a randomized clinical trial, 153 undergraduate students from a large public university in the United States completed three phases of pre-assessment, intervention, and post-assessment over six weeks during the COVID-19 pandemic. During the intervention, participants were randomized to receive animated videos of DBT skills for 14 consecutive days. All participants received ecological momentary assessments of mood, emotion management self-efficacy, and emotional tolerance four times a day. The study found that negative emotions significantly decreased and positive emotions significantly increased before and after watching the videos. There was a significant interaction between time and conditions in the development of emotional tolerance. Compared to the first two weeks, participants in the control group rated their emotions as more intolerable in the third and fourth weeks, whereas participants in the intervention group did not rate their emotions as more intolerable (Rizvi et al., 2022). This proved that DBT techniques can help college students avoid a decline in mental health and that this simple, highly scalable intervention could expand the scope of available mental health treatment.

In a Chinese study, 1,607 college students in Wuhan were asked about their mental health, exercise-related lifestyle, and other issues. The Depression, Anxiety, and Stress Scale (DASS-21) was used to evaluate their mental health. The results showed that lower DASS-21 scores were significantly associated with regular exercise, maintaining exercise habits during the COVID-19 pandemic, exercising more than 1–2 times per week, exercising for ≥1 h, and taking ≥2,000 steps (Deng et al., 2020). This proved that mental status is significantly related to regular exercise and adequate exercise time. In a similar article, a longitudinal survey of 66 college students during the peak of the COVID-19 epidemic in China showed that COVID-19 had a direct negative impact on general sleep quality. In contrast, COVID-19 mediated general negative emotions, stress, anxiety, and sleep quality. In addition, physical activity directly alleviated general negative emotions, with the greatest effect when physical activity was approximately 2,500 METs per week (Zhang et al., 2020).

In a study of all medical students in Jordan, participants self-reported increased levels of negative emotions, such as anxiety, and decreased levels of positive emotions. Nearly half of the participants reported that social media was the primary source of COVID-19 information, with a significant reduction in emotional distress after long-term use of social media for COVID-19 information (Kheirallah et al., 2021). This was evidence that social media has a potentially positive effect on mitigating negative emotions.

Of the 18 included studies, three suggested that improving sleep quality could indirectly improve stress and anxiety in students during COVID-19 (Zhang et al., 2020; Liu et al., 2021; Song et al., 2022). In a study examining the relationship between perceived stress and depression in medical students during the COVID-19 pandemic and the mediating role of insomnia in this relationship, researchers used the Perceived Stress Scale (PSS), Insomnia Severity Index (ISI), and Patient Health Questionnaire 9 (PHQ-9) to measure perceived stress, insomnia, and depression levels. Results showed that perceived stress was significantly associated with depression. Insomnia mediated the relationship between perceived stress and depression. The indirect effect of insomnia on perceived stress was significant (Liu et al., 2021). This demonstrates that depression in medical students can be effectively reduced by improving sleep quality and relieving perceived stress. In a similar study of 666 medical students in China, anxiety was significantly associated with problematic smartphone use and sleep disturbances during the COVID-19 pandemic. Problematic smartphone use not only directly affected anxiety but also had a significant indirect effect on anxiety through sleep disturbance. Using sleep disturbance as a mediator, a significant reduction in the path coefficient of problematic smartphone use on anxiety was observed. The importance of promoting sleep health to reduce anxiety should be emphasized (Song et al., 2022).

HP Lovecraft, in “Supernatural Horror in Literature” (1927), H.P. Lovecraft wrote: “The oldest and strongest emotion of mankind is fear, and the oldest and strongest kind of fear is fear of the unknown” (Joshi and Schultz, 2001; p. 255). Therefore, among the 18 included studies, five were of the opinion that we should actively face COVID-19 and that increasing its understanding through various means would help reduce students’ stress and anxiety (Banstola et al., 2021; Kheirallah et al., 2021; Simegn et al., 2021; Yildirim et al., 2021; Chan et al., 2022).

The study of 202 medical students in Hong Kong about their mental health status, stress coping strategies, and their relationship proved that respondents who adopted an approach strategy had higher Ryff scores than those with avoidant coping strategies, suggesting that approach strategies are more effective in stress management than the more dysfunctional avoidant strategies (Chan et al., 2022). Similarly, Banstola et al. (2021) suggested that adopting adaptive coping strategies helps reduce pandemic-related mental health problems. Kheirallah et al. (2021) suggested that students’ use of social media to increase access to COVID-19 information had a significant effect on reducing emotional distress. Simegn et al. (2021) found that the mental health situation can be improved by the provision of adequate and accurate information and by increasing the self-efficacy of students. Yildirim et al. (2021) indicated that this would be an effective method to provide training to nursing students, to change their negative attitudes, increase their awareness of COVID-19-related death anxiety, improve their coping skills for dealing with death anxiety, and reduce the burden of anxiety. Table 2 provides a summary of the studies included in this review. Across all works, data were derived from assessments of 44,014 participants. Of these, 14,790 were men (33.60%), 26,547 were women (60.32%), and 2,677 were undetermined (6.08%). The number of participants ranged from 40 to 29,663. The studies included 32,973 medical students and 11,041 non-medical students.

**Table 2:** Summary of this systematic review.

Sample size (*n*)		Gender(*n*)	
<100	3	Men only	0
101–500	7	Women only	1
501–1,000	3	Men and Women	17
1,001–2,000	2	Undetermined	0
2,001–4,000	1	Region(*n*)	
>4,001	2	Americas	2
Participants(*n*)		Asia	11
College student	18	European	4
Medical student	9	Africa	1
Non-medical student	9	Australia	0
Intervention type(*n*)			
Improving understanding			5
Improving sleep quality			3
Physical activity			3
Other			8

The research included in this review covers three years (01/2020–12/2022). The participants in these studies were college students. According to the country distribution of the participants, Asia (11), the Americas (2), Europe (4), Australia (0), and Africa (1). From the perspective of the interventions used, the most common effective interventions were sustained physical exercise at a certain intensity for a period of time, maintaining good sleep quality, improving understanding of COVID-19 through multimedia, and facing COVID-19 with more positive attitudes and behaviors. In addition, regular watching of DBT skill videos may help college students avoid mental health issues.

## Discussion

The COVID-19 pandemic has resulted in substantial global mental health challenges, such as increased levels of anxiety and depression symptoms (Brooks et al., 2020; Holmes et al., 2020), along with significant variation in anxiety and depression symptoms among residents of different countries (Ding et al., 2021a,b). In this review, 18 studies on anxiety and stress levels among students during the COVID-19 pandemic were identified, while also listing the effects of different interventions. The 11 studies that did not explicitly report anxiety among participants all reported higher rates of anxiety among participants; in the seven studies where anxiety was explicitly reported, the average anxiety rate was 77.99%. Hence, this shows the high level of anxiety among students due to COVID-19.

Studies have shown that students’ anxiety about COVID-19 stems not only from concerns about their health but also from the health of those around them (Al-Kumaim et al., 2021). In addition, academic pressure and employment prospects during this period are also major causes of anxiety for students (Sundarasen et al., 2020). In particular, female, rural, low-income, and academically underperforming students are more likely to suffer from psychological distress (Lee et al., 2021). These key findings are of great concern given that mental health is strongly linked to student well-being, academic performance, and employment rates. During the pandemic, student mental health is in crisis and necessitates increased attention and intervention.

The primary focus of this study was to identify more effective interventions. Four of the 18 included studies reported significant reductions in student stress and anxiety. The selected interventions included: (1) Watching DBT skill videos. The results of this study showed that the simple animated DBT skill videos were easily accepted by most of the participants; the intervention showed promising results in reducing negative emotions at the time and preventing students from experiencing increased distress (i.e., finding their emotions more unbearable) as the semester progressed. This intervention is also very easy to implement (Rizvi et al., 2022). (2) 2,500 METs of physical activity per week. The results of this study showed that the persistence of COVID-19 may reduce people’s sleep quality, thereby significantly increasing their negative emotions; maintaining regular exercise can help alleviate this state of mind, and engaging in physical activity (PA) of 2,500 METs per week is the most effective optimal load (Zhang et al., 2020). (3) Extending the use of social media to gain a better understanding of COVID-19 has been shown to reduce students’ fear of COVID-19 and improve their negative mental state (Deng et al., 2020; Kheirallah et al., 2021).

Three of the 18 selected studies showed that due to the persistence of COVID-19, students have experienced sleep disturbance or insomnia, and the continuation of this phenomenon will lead to stress and anxiety. Improving sleep quality can indirectly help with these symptoms (Zhang et al., 2020; Liu et al., 2021; Song et al., 2022). In addition, increasing physical activity is not only beneficial in improving stress and anxiety caused by COVID-19, but it also happens to be an effective way to improve sleep quality (Piercy et al., 2018 Zhang et al., 2020). There is now a consensus that appropriately heightened physical activity can help relieve stress and anxiety, promote blood circulation, relax muscles, and improve sleep quality; multi-component exercise has a significant effect on improving people’s sleep quality and physical fitness (Yang, 2019 Ai et al., 2022).

By synthesizing the results of previous studies, we have drawn a comprehensive summary that includes effective intervention methods and combined these with the current situation. Therefore, we have been able to clearly identify the most effective intervention measures. At this stage, COVID-19 is no longer an unfamiliar disease. As a result, simply getting more information about COVID-19 through social media no longer seems to be an effective way to reduce the resulting stress and anxiety. Although watching DBT skill videos is considered an effective intervention, due to the relatively small number of similar studies, it is not yet possible to provide sufficiently strong evidence. After a comprehensive comparison, it can be found that appropriate physical activity can not only directly reduce the stress and anxiety caused by COVID-19 but can also indirectly relieve these by improving sleep quality. Therefore, this can be regarded as the most effective means of intervention at this stage. Proper physical exercise can also help improve our body’s immune system, increase the speed of antibody production, and improve the ability to resist viral attacks (Atzrodt et al., 2020). Therefore, more research in the future can address the need to improve sleep quality through physical activity to alleviate the perceived stress caused by COVID-19 and effectively reduce students’ anxiety.

The main focus of this paper is to examine the effects of various intervention methods applied during the COVID-19 pandemic. However, there are still some limitations. Firstly, many of the included studies in the literature were cross-sectional studies, meaning that a temporal causal relationship between mental health and stress-coping strategies could not be established. The reason for this is that, although there is a large amount of research on psychological problems caused by COVID-19 at this stage, fewer articles can be retrieved with the inclusion of the keywords “student” and “intervention.” Therefore, future research should focus more on the impact of COVID-19 on students’ mental states and potential solutions. Second, all included studies used self-report methods to assess anxiety and depression scores, and mental health counselors were not asked to evaluate the psychological status of the participants as a form of third-party verification. Although this method simplifies the difficulty of obtaining data on the mental state of subjects, it is highly subjective and may be affected by social desirability bias. However, since all papers considered found a higher rate of anxiety in the study groups, the results are generally reliable. In addition, the participants included in the literature are all college students, so it is impossible to conclude whether the interventions applicable to college students are also applicable to students in other age groups. Similarly, the research subjects included in the literature are mostly medical students. Since these students have medical knowledge and skills, in addition to certain characteristics, it is impossible to conclude whether the interventions applicable to medical students are also applicable to non-medical students.

## Conclusion

This study provides a comprehensive look at solutions to student anxiety during COVID-19. By comparing different interventions, we found that appropriate physical activity has unique benefits that not only directly reduce the stress and anxiety caused by COVID-19 but also further alleviate these feelings by improving sleep quality. Currently, the persistence and impact of COVID-19 on daily life present students with unprecedented psychological challenges. Appropriate physical activity, as a comprehensive intervention that provides both a physical and mental health response, was found to be the most effective intervention for students. Of course, we must also acknowledge the limitations of this study and recognize that physical activity, while effective, is not the only solution. A combination of other interventions is also critical when dealing with the stress and anxiety of COVID-19. Therefore, in the future response to the epidemic, we should continue to explore, research, and promote the combination of more targeted physical activities and other interventions to better help students cope with the challenges they may face during COVID-19.

## Data availability statement

The datasets presented in this study can be found in online repositories. The names of the repository/repositories and accession number(s) can be found in the article/supplementary material.

## Author contributions

JW, GK, HL, and YK conceptualized and designed the original study, from which the data for the analyses presented here were obtained. JW, GK, and YK completed data collection, data analysis, and initial writing. JW, GK, HL, XH, MM, AS, and YK participated in writing and commenting on the manuscript and drafted, edited, and approved its final version. All authors read and agreed to the published version of the manuscript.

## Funding

This research was supported by the Ministry of Higher Education Malaysia for the Fundamental Research Grant Scheme (FRGS) with project code: FRGS/1/2020/SKK06/USM/03/13.

## Conflict of interest

The authors declare that the research was conducted in the absence of any commercial or financial relationships that could be construed as a potential conflict of interest.

## Publisher’s note

All claims expressed in this article are solely those of the authors and do not necessarily represent those of their affiliated organizations, or those of the publisher, the editors and the reviewers. Any product that may be evaluated in this article, or claim that may be made by its manufacturer, is not guaranteed or endorsed by the publisher.
